# Variables associated with unhealthy diet among Portuguese adults: a population-based logistic regression study

**DOI:** 10.3389/fnut.2026.1832783

**Published:** 2026-06-09

**Authors:** Ana Muriel Assunção, Rosália Páscoa, Pedro Rodrigues, Andreia Teixeira

**Affiliations:** 1 MEDCIDS, Faculty of Medicine, University of Porto, Porto, Portugal; 2 RISE-Health, Department of Community Medicine, Information and Health Decision Sciences, Faculty of Medicine, University of Porto, Porto, Portugal; 3 USF Homem do Leme, Porto Ocidental Primary Health Care Center, Porto, Portugal

**Keywords:** dietary patterns, lifestyle, logistic regression, Portugal, unhealthy diet

## Abstract

**Background:**

Noncommunicable diseases, responsible for most global deaths, are closely linked to unhealthy lifestyles, particularly a poor diet. There has been an important shift in dietary habits, deviating from national and international recommendations. In contrast, the adult Portuguese population appears to overestimate the healthfulness of their diet, highlighting the need to better understand these behaviors and objective associations.

**Methods:**

This study used weighted multiple logistic regression to identify associated variables with unhealthy diet among 891 Portuguese adults from a cross-sectional, population-based survey conducted in 2019. A healthy diet was defined by regular meals, adequate fruit and vegetables intake, and moderate alcohol consumption, according to established public health guidelines.

**Results:**

Non-smokers were less likely to have an unhealthy diet [OR = 0.21 (0.07–0.53)], while screen users were more likely [OR = 2.36 (1.12–5.11)]. The model showed moderate discrimination, AUC = 0.62.

**Conclusion:**

Smoking and screen-related behaviors independently were associated with unhealthy diet, underscoring the need for integrated public health strategies targeting multiple lifestyle risks to foster healthier habits.

## 1 Introduction

Noncommunicable diseases (NCDs) are a major public health challenge, which accounted for approximately 1.80 billion global disability-adjusted life-years (DALYs) in 2023 (1, 2). The burden of NCDs is associated with ageing populations and the globalization of unhealthy lifestyles, such as unhealthy diets, physical inactivity, smoking and harmful alcohol consumption (1, 3). These lifestyles may manifest through metabolic risk factors, whose attributable DALYs have exhibited a consistent increase in previous systematic analyses encompassing 204 countries and territories based on the Global Burden of Disease Study (GBD), despite a decline in age-standardized DALY rates (2, 4).

Over the past decades, there has been a significant change in dietary habits, including an increased consumption of ultra-processed foods and beverages, associated with higher caloric intake, higher sweetener, sodium, unhealthy fats and additives consumption, and lower essential nutrients intake (5–7). The consumption of these foods may increase the risk of several NCDs, including overweight and obesity, metabolic syndrome, elevated waist circumference and reduced HDL-cholesterol levels (8, 9). On the contrary, dietary patterns rich in fiber and nutrients, and low in proinflammatory foods, such as the Mediterranean diet, have demonstrated beneficial effects in the prevention and management of NCDs (10, 11).

In Portugal, according to the most recent national data available from the National Food, Nutrition, and Physical Activity Survey (2015–2016), an increased consumption of meat, fish, eggs, and dairy products was observed, alongside a reduced fruit, vegetable, and legume intake, deviating from the recommendations outlined in the Portuguese Food Wheel guide and the World Health Organization (WHO) guidelines (7, 12). Soft drinks were the second most consumed beverage, with an average intake of 88 g/day, particularly among adolescents (12). Despite these patterns, many individuals overestimate the healthfulness of their diet. A previous cross-sectional study found that only 14% of Portuguese adults met the criteria for a healthy diet, whereas 87% “totally agreed” and 12% “partially agreed” that their diet was healthy (13).

Recent findings from the GBD 2023 study reinforce the major contribution of unhealthy dietary patterns to mortality and DALYs in Portugal. In 2023, inadequate dietary habits were associated with approximately 7.9% of all deaths and 5.3% of DALYs in the Portuguese population, while metabolic conditions strongly linked to diet - namely high body mass index (BMI), elevated plasma glucose, and hypertension - remained among the leading contributors to disease burden (2).

Considering this prominent lifestyle pattern at both national and international levels, the need to identify populations at risk becomes evident, to implement mitigation strategies and thereby reduce the burden of NCDs. The management and prevention of NCDs sets its foundations on multiple levels - individual, society, country, and global, such as the Global Action Plan for the prevention and control of noncommunicable diseases defined by the WHO (14, 15). At the societal level, the detection and screening of risk factors play a very important role (1, 14). This highlights the need to study what might objectively be associated with unhealthy eating behaviors.

Given the importance of accurately identifying factors associated with unhealthy diet, logistic regression offers a powerful statistical tool to analyze the association between binary outcomes and multiple predictors, adjusting for confounders and estimating the strength of associations via odds ratios (16, 17). Previous studies have applied logistic regression models to examine factors associated with healthy dietary behaviors (18–20). Adherence to physical activity and screen-time recommendations has been consistently associated with healthier dietary patterns, including increased fruit and vegetable intake and reduced sugar-sweetened beverage consumption (18–20).

The present study aimed to identify the variables associated with an unhealthy diet among Portuguese adults using weighted multiple logistic regression analysis. This might contribute to a potentially important tool to design public health strategies, thereby contributing to the management of NCDs.

## 2 Materials and methods

### 2.1 Study design and participants

The present study was derived from the database of Patients’ Perspectives about Lifestyle Behaviors and Health in the Context of Family Medicine: A Cross-Sectional Study in Portugal; a previously published study conducted in a representative sample of the mainland Portuguese adult general population (≥20 years), through a structured questionnaire applied in person at the participant’s home (13). The exclusion criteria included having a cognitive or physical impairment limiting the ability to participate in the interview, residing in a collective dwelling, inability to speak or understand the Portuguese language and refusing to give informed consent for study participation (13, 21). Individuals residing in collective dwellings were excluded once their lifestyle behaviors may be influenced by institutional or shared conditions.

Sampling followed a stratified design by NUTS II regions (nomenclature of territorial units for statistical purposes), in which random starting points were selected for each unit, with a probability proportional to the population size of the respective NUTS. There were quotas for gender, age, and region of residence, and the interviews were conducted in all district capitals. To select the participants, the random route sampling method and the last birthday method were applied. Respectively, each street, door number and floor was chosen randomly, and, in each household, was selected the individual whose birthday was more recent (13).

The sample size of the present study comprises 891 participants, which corresponds to a 3.2% margin of error, assuming a 95% confidence level, a conservative scenario and an infinite population.

The study was conducted in accordance with the guidelines of the Declaration of Helsinki and approved by the Ethics Committee of the São João Hospital Center/Faculty of Medicine of the University of Porto (protocol code: 140–18 and date of approval: 14 December 2018) (13).

### 2.2 Data collection

Data were collected between January and April 2019. The interviewers were trained to clarify the meaning of each question and were monitored by a data collection supervisor. Total completion took between 20 and 25 min. The questionnaire was specifically designed for the initial study, since there was, at the time, no known validated questionnaire. It covered 3 areas: 1 - health status, including self-perception of health and health problems in the last 12 months; 2 - lifestyle, such as diet and alcohol consumption; and 3 - sociodemographic data, including sex, gender, marital status, highest level of education completed and others (13).

A healthy diet was defined, in alignment with established global public health recommendations supported by the WHO, as whenever the participant concomitantly ate two to three main meals per day, two to six portions of fruit per day, two or more portions of vegetables or salads per day, and had a moderate consumption of alcohol (≤1 drink/day for women and men ≥65 years, and ≤2 for younger men) (7, 13, 22–24).

### 2.3 Statistical analysis

Data were analyzed using the Statistical Package for the Social Sciences (SPSS, version 29.0.2.0) and the R Studio software. Categorical variables were expressed as absolute and relative frequencies, n (%). Normally distributed continuous variables were described by the mean and standard deviation, M (sd), and non-normally distributed continuous variables by the median and interquartile interval, Med [Q_1_; Q_3_], where Q_1_ is the first quartile, and Q_3_ is the third quartile. The normality of the distributions was verified by visualization of the respective histograms.

The sample was randomly divided into learning (70%) and testing (30%) sets, with the aim of reducing overfitting and allowing assessment of the model’s generalization ability. Firstly, weighted univariate logistic regressions were performed in the learning group (LG). Healthy diet was determined as the dependent variable (“yes” as the reference category) and demographic (gender, age, marital status, BMI, highest level of education completed and main occupation), health (self-perceived general health status, cardiovascular problems, metabolic problems, mental health problems, osteoarticular pain and none), and lifestyle (smoking, regular physical activity and screen activity) as independent variables. Subsequently, variables with *p* ≤ 0.20 were entered into a weighted multiple logistic regression model (initial model), and, though a stepwise procedure, used as an exploratory method, were retained only statistically significant variables (*p* ≤ 0.05) (final model). Results were reported as odds ratios (OR) and 95% confidence intervals (95% CI) for variables associated with an unhealthy diet.

Model performance was evaluated in terms of discrimination and calibration, using predicted probabilities, the receiver operating characteristic (ROC) curve, the area under the curve (AUC), and the Hosmer–Lemeshow goodness-of-fit test. Discrimination metrics included sensitivity, specificity, positive predictive value (PPV), and balanced accuracy, calculated at selected probability thresholds.

## 3 Results

**Figure 1:**
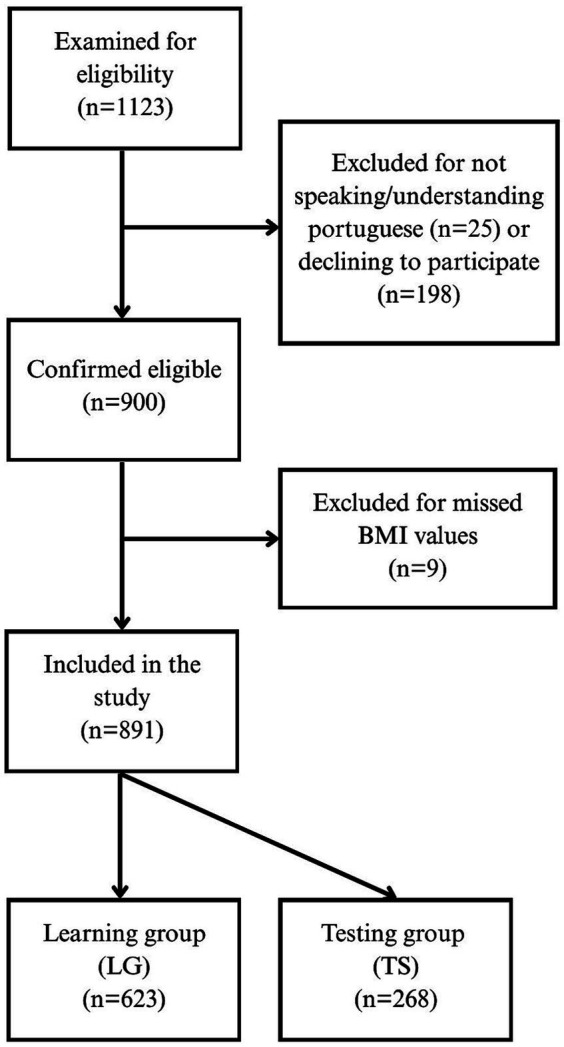
Participants’ flow diagram.

During the interviewing process, for the initial published investigation, 223 individuals were considered not eligible; 25 did not speak or understand Portuguese, while 198 refused to participate, resulting in 900 participants who answered the questionnaire (13). In the subsequent investigation, 9 individuals were excluded due to missing anthropometric data (BMI), leading to 891 participants (21). Finally, participants were segmented into learning and testing groups (Figure 1).

To provide appropriate context for the findings of the present study, we first summarize the most relevant overall results of the previous study conducted in this representative sample of the Portuguese adult population (21). Participants´ general characteristics and habits are presented in Table 1 and Supplementary Table 1. In the total sample, 53.6% of participants were female, with a mean age of 51.8 years (SD = 18.1). Most respondents were Portuguese (95.4%), married (56.2%), had completed lower or upper secondary education (62%), and were professionally active (63.3%). The mean BMI was 25.8 (SD = 4.0). Most participants reported their self-perceived health status as good or very good (55.1%). Osteoarticular pain in the previous 12 months was reported by 38.6% of participants, while 39.5% reported none of the health problems assessed. Regular physical activity was reported by 28.7, 25.4% were current smokers, and 35.7% engaged in screen-based activities. Only 13.9% met the criteria for a healthy diet.

**Table 1:** Participants’ characteristics in the total sample (TS), learning group (LG), and testing group (TG).

Variables		TS (*n* = 891)	LG (*n* = 623)	TG (*n* = 268)
Gender, *n* (%)	Female	478 (53.6)	341 (54.7)	137 (51.1)
	Male	413 (46.4)	282 (45.3)	131 (48.9)
Age, years, M (sd)		51.8 (18.1)	51.8 (18.0)	51.7 (18.3)
Nationality, *n* (%)	Portuguese	850 (95.4)	594 (95.3)	256 (95.5)
	Foreign	41 (4.6)	29 (4.7)	12 (4.5)
Marital status, *n* (%)	Married	501 (56.2)	340 (54.6)	161 (60.1)
	Others	390 (43.8)	283 (45.4)	107 (39.9)
Weight, kg, M (sd)		71.7 (12.7)	71.8 (12.7)	71.4 (12.6)
Height, cm, M (sd)		166.6 (9.5)	166.7 (9.4)	166.4 (9.8)
BMI, M (sd)		25.8 (4.0)	25.8 (4.1)	25.7 (3.6)
Education level completed, *n* (%)	1st cycle or less	224 (25.1)	149 (23.9)	75 (28.0)
	2nd or 3rd cycle	258 (29.0)	196 (31.5)	62 (23.1)
	Secondary	294 (33.0)	197 (31.6)	97 (36.2)
	Higher education	115 (12.9)	81 (13.0)	34 (12.7)
Main occupation, *n* (%)	Active	564 (63.3)	393 (63.1)	171 (63.8)
	Non-Active	327 (36.7)	230 (36.9)	97 (36.2)
Self-perceived health status, *n* (%)	Very good or good	491 (55.1)	334 (53.6)	157 (58.6)
	Reasonable	322 (36.1)	235 (37.7)	87 (32.5)
	Bad or very bad	78 (8.8)	54 (8.7)	24 (9.0)
Health problems in the last 12 months, *n* (%)	Cardiovascular	276 (31.0)	187 (30.0)	89 (33.2)
	Metabolic	213 (23.9)	145 (23.3)	68 (25.4)
	Mental health	171 (19.2)	121 (19.4)	50 (18.7)
	Osteoarticular pain	344 (38.6)	245 (39.3)	99 (36.9)
	None	352 (39.5)	238 (38.2)	114 (42.5)
Regular physical activity, *n* (%)		256 (28.7)	184 (29.5)	72 (26.9)
Smokers, *n* (%)		226 (25.4)	159 (25.5)	67 (25.0)
Screen activity, *n* (%)		318 (35.7)	214 (34.3)	104 (38.8)
Healthy diet, *n* (%)^a^		124 (13.9)	88 (14.1)	36 (13.4)

Sociodemographic, health and lifestyle data are shown. Categorical variables are expressed as n (%), normally distributed continuous variables as Mean (sd), and non-normally distributed continuous variables as Median [Q1; Q3].

a A healthy diet was defined as whenever the participant concomitantly ate two to three main meals per day, two to six portions of fruit per day, two or more portions of legumes or salads per day, and had a moderate consumption of alcohol (≤1 drink/day for women and men ≥65 years, and ≤2 for younger men).

Results from the univariate logistic regressions are presented in Supplementary Table 2. In the final multiple logistic regression model (Table 2), two variables remained significantly associated with unhealthy eating habits. Non-smokers were less likely to have an unhealthy diet than smokers [OR = 0.21 (0.07; 0.53), *p* = 0.002], while participants with screen-related activities were more likely to do so [OR = 2.36 (1.12; 5.11), *p* = 0.026].

**Table 2:** Learning group’s weighted multiple logistic regression analysis to identify independent factors associated with unhealthy diet.

Variable		Initial model		Final model	
		OR [95% CI]	*p*-value	OR [95% CI]	*p*-value
Gender	Female	*Reference*			
	Male	1.86 [0.91; 3.84]	0.089		
Smoker	Yes	*Reference*		*Reference*	
	No	0.25 [0.09; 0.65]	**0.007**	0.21 [0.07; 0.53]	**0.002**
Screen Activity	Absent	*Reference*		*Reference*	
	Present	2.49 [1.17; 5.48]	**0.020**	2.36 [1.12; 5.11]	**0.026**
Osteoarticular pain	No	*Reference*			
	Yes	0.68 [0.34; 1.37]	0.280		

In the final model, non-smokers remained negatively associated with presenting an unhealthy diet, while presenting excessive screen activity was positively associated. OR, odds ratio; 95% CI, 95% confidence interval; Results considered significant at the 5% level. Initial model and final model are presented. The bold values represent statistically significant variables (*p* ≤ 0.05).

To evaluate the discriminative ability of the logistic regression model in the learning data, predicted probabilities were used to generate receiver operating characteristic (ROC) curves across different probability cutoffs of 0.3, 0.4, and 0.5. Sensitivity, specificity, positive predictive value (PPV), and overall accuracy were calculated for each cutoff. The threshold of 0.4 provided the best balance between sensitivity (0.56) and specificity (0.73), with a positive predictive value of 0.93 and a balanced accuracy of 0.64. The area under the ROC curve (AUC) was 0.66, indicating a moderate discriminative performance.

Calibration was evaluated using the Hosmer–Lemeshow goodness-of-fit test and a calibration plot comparing predicted and observed probabilities. The Hosmer–Lemeshow test indicated some miscalibration (*p* < 0.001), although the overall calibration curve showed reasonably close agreement between predicted and observed probabilities.

**Figure 2:**
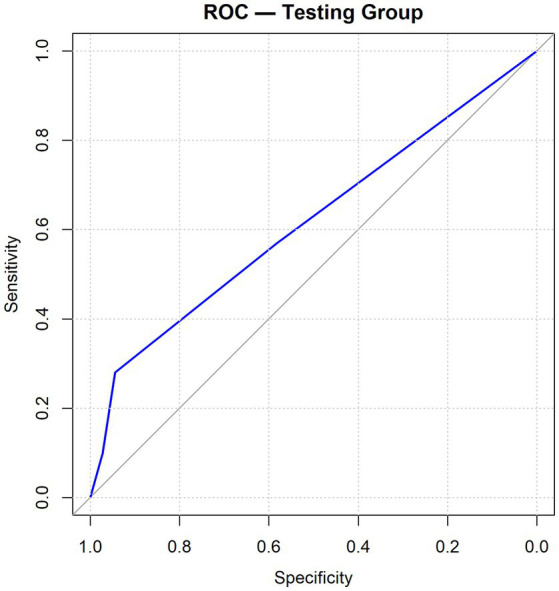
Receiver operating characteristic (ROC) curve in the testing dataset for prediction of unhealthy diet. Sensitivity is plotted on the *y*-axis and specificity on the *x*-axis. The area under the curve (AUC) of 0.62 confirms consistent moderate predictive performance.

When applied to the independent test dataset, the model achieved a similar AUC = 0.62, confirming consistent, moderate predictive performance (Figure 2), similar to that observed in the learning data. Using the previously defined cutoff of 0.4, the model’s classification performance was summarized by the confusion matrix and related metrics (Table 3).

**Table 3:** Performance metrics of the logistic regression model in the testing dataset.

Metric	Estimate
AUC	0.618
Sensitivity	0.569
Specificity	0.583
Positive predictive value (PPV)	0.898
Negative predictive value (NPV)	0.174
Accuracy	0.571
Balanced accuracy	0.576

**Figure 3:**
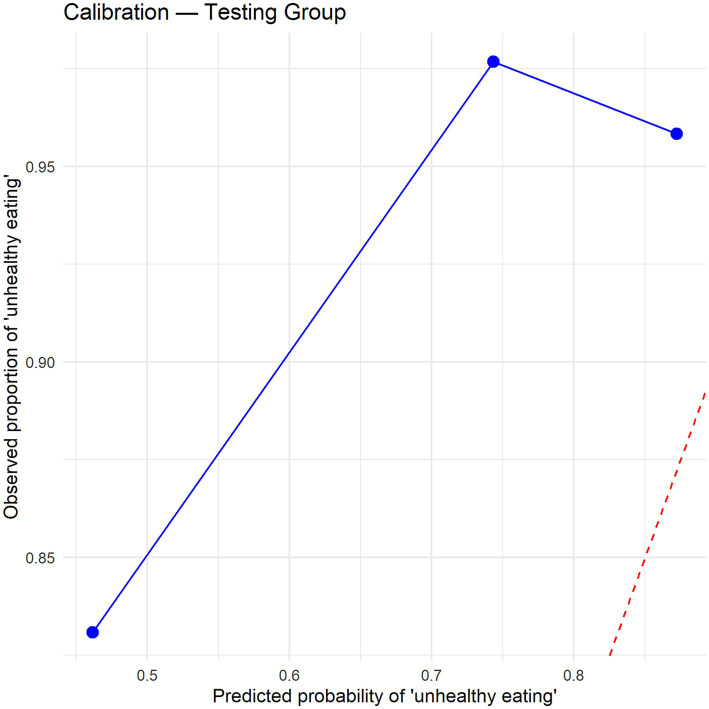
Calibration plot comparing the observed proportion of unhealthy eating (*y*-axis) with the predicted probability of unhealthy eating (*x*-axis) in the testing dataset. Predicted and observed probabilities show an approximately linear relationship, indicating acceptable agreement.

At the 0.4 cutoff, the model showed a sensitivity of 0.57 and specificity of 0.58, reflecting a balanced accuracy of 0.58. The positive predictive value (PPV) was high (0.90), suggesting that most cases predicted as ‘unhealthy’ were indeed unhealthy. Despite modest overall accuracy (0.57), this performance aligns with the study’s goal of prioritizing detection of unhealthy eating habits, even at the expense of a higher false-positive rate. Calibration analysis showed limited fit (Hosmer–Lemeshow *p* < 0.001) but an approximately linear relationship between predicted and observed probabilities, indicating acceptable agreement (Figure 3).

The model demonstrates consistent generalization from training to testing. Its capacity to correctly identify individuals with unhealthy eating habits, despite limited overall accuracy and predictive performance, supports its potential usefulness for public health screening where minimizing false negatives is a priority.

## 4 Discussion

A previous study found that Portuguese people overestimated the healthfulness of their diet, while only 13.9% of participants met the criteria for a healthy diet, underscoring the need to deepen our knowledge about lifestyle, diet and, associated objective variables (13).

Other previous studies conducted in Portugal suggest the existence of an association between demographic, social, economic, and behavioral factors and the presence of healthy or unhealthy dietary patterns (Mediterranean diet, EAT-Lancet Planetary Health Diet, sustainable healthy diets, consumption of ultra-processed food and beverages, among others) (25–30). Being female, compared to being male, is significantly associated with healthier dietary patterns (27, 29, 30), as is older age when compared to younger individuals (26, 29, 30). Living with a partner and residing in less urbanized areas are also associated with healthier diets/less consumption of unhealthy foods (26, 29). Among adults, higher education is associated with greater consumption of healthy foods, but also with higher consumption of ultra-processed foods (26, 28, 29). Experiencing food insecurity and having a low income are associated with less healthy dietary choices (27, 28, 30). However, most of the recent studies are based on the National Food, Nutrition and Physical Activity Survey from 2015 to 2016. Further research is needed to monitor changes in dietary patterns over time and the resulting generational shifts.

The present population-based study identified smoking and screen-related behaviors as independent factors associated with unhealthy diet among Portuguese adults. This aligns with previous evidence suggesting that unhealthy behaviors tend to cluster within broader lifestyle risk patterns (2, 4, 13).

Several studies associate adult smokers with having poor diets, such as presenting altered nutrient intake, higher fast-food/fried food consumption, white meat consumption, decreased fruit, vegetables and dairy products intake, and higher alcohol consumption, both on international (31–35) and national (36, 37) levels. These effects may be due to a diminished food intake and a shift in dietary preferences, relating to lower health consciousness and nicotine’s effects on decreasing appetite and increasing metabolic rate, resulting in smaller meal portions, less snacking, but higher cravings for fast-food and high-fat foods. It also relates to changes in hormones, such as ghrelin or leptin, and altered hedonic value of food (31, 35). Evidence also suggests that there might be an alteration of neurophysiological mechanisms, leading to a desensitization to natural food-related reward systems in the brain (35).

Similarly, screen time, through television or digital device use, was linked to the consumption of unhealthy foods and an increased dietary intake, both in adults and younger people. This might be related to increased snacking, greater exposure to food advertising, leading to hunger and craving, and other non-advertising effects, such as the distraction factor and impaired memory formation (38–43).

Considering that the data collection of the present study predates 2020 and the COVID-19 pandemic, it is safe to hypothesize that several lifestyle factors might have suffered changes that prevailed through time, altering the population’s profile. For instance, given the increase of time spent indoors, including online school and remote work, during the beginning of this decade, time spent on screen activities might have increased (44). On the other hand, smoking and alcohol consumption might have had different possible trajectories. Due to an increase of stress through this difficult period, the use and consumption of these substances might have increased, however the decrease of parties and social gatherings in general, and higher health consciousness might have contributed to their reduction (45, 46). Bearing this in mind, findings describe associations within the studied period and caution is needed to extrapolate to the current context.

Given that the data analyzed in the present study were derived from previously published investigations, the limitations inherent to those studies must be acknowledged. The main limitations include the cross-sectional design, which precludes causal inference, and the reliance on self-reported data (13, 21). Potential recall bias should therefore be considered, even though some questions intentionally referred to short recall periods (13, 21). It should also be noted that the definition of the variable “healthy diet” was not based on validated indices, but rather on established global public health recommendations. Additionally, it might not be sufficiently comprehensive to capture all the complexity of dietary patterns and current evolution, including the consumption of ultra-processed foods and beverages, thus misclassification bias should be considered. In addition, individuals residing farther from district capitals may be under-represented, and the study sample was restricted to residents of mainland Portugal (13, 21). Finally, potentially relevant socioeconomical confounders, such as income and food access, were not included in the analysis. Future studies should incorporate these variables to improve model robustness.

Some instability and overfitting of the model can be considered in the present study, since a stepwise variable selection was used, however, it showed consistency from training to testing. The model showed modest discriminatory ability (AUC = 0.62) and limited calibration, however as it is not intended to precise individual prediction, it may have potential in identification of population-level associations and risk patterns. As the study sample was based on a representative sample of the mainland Portuguese adult population, these findings may reflect behavioral patterns associated with unhealthy eating habits at the population level in Portugal (13, 21). Consistent model performance provides acceptable external validity and reinforces the public health relevance of the observed associations.

## 5 Conclusion

In the present study, smoking and screen-related behaviors were associated with unhealthy diet among mainland Portuguese adults. These observations align with previously mentioned studies and highlight the need for integrated strategies tackling multiple lifestyle risks, contributing to the management of NCDs. More individual, society, country, and global interventions must be created to promote healthier diets. The observed associations might help design public health strategies to assess and improve populations dietary habits.

## Data Availability

The datasets supporting the conclusions of this study are not publicly available but will be made available from the corresponding author upon reasonable request.
