# Supplementary material for: Variables associated with unhealthy diet among Portuguese adults: a population-based logistic regression study
Source: Front Nutr. 2026 Jun 9;13:1832783. doi: 10.3389/fnut.2026.1832783 (PMC13286782; doi:10.3389/fnut.2026.1832783)
Supplement: Supplementary file 1 [file Table_1.docx]

Supplementary Material

# Supplementary Tables

**Supplementary Table 1.** Participants’ eating and alcohol drinking habits in the total sample (TS), learning group (LG) and testing group (TG). Categorical variables are expressed as n (%), normally distributed continuous variables as Mean (sd), and non-normally distributed continuous variables as Median [Q1; Q3].

| Variables | | | TS | LG | TG |
| --- | --- | --- | --- | --- | --- |
|  |  |  | (n=891) | (n= 623) | (n=268) |
| Number of main meals per day, Med [Q1, Q3] | | | 3.0 [2.0;3.0] | 3,0 [3.0;3.0] | 3.0 [2.0;3.0] |
| Number of intermediate meals per day, Med [Q1, Q3] | | | 2.0 [1.0; 2.0] | 2.0 [1.0;2.0] | 1.0 [1.0;2.0] |
| Consumed in the main meals the day before, n (%) | | *Milk, yogurt or cheese* | 656 (73.6) | 461 (74.0) | 195 (72.8) |
|  |  | *Vegetable soup* | 592 (66.4) | 421 (67.6) | 171 (63.8) |
|  |  | *Bread* | 753 (84.5) | 521 (83.6) | 232 (86.6) |
|  |  | *Beef* | 697 (78.2) | 489 (78.5) | 208 (77.6) |
|  |  | *Fish* | 473 (53.1) | 333 (53.5) | 140 (52.2) |
|  |  | *Potatoes, rice or pasta* | 726 (81.5) | 504 (80.9) | 222 (82.8) |
|  |  | *Beans or chickpeas* | 249 (27.9) | 182 (29.2) | 67 (25.0) |
|  |  | *Cakes, cookies, chocolates or desserts* | 360 (40.4) | 244 (39.2) | 116 (43.3) |
|  |  | *Soft drinks, with or without gas* | 193 (21.7) | 141 (22.6) | 52 (19.4) |
|  |  | *Natural juices, made from fresh fruit* | 166 (18.6) | 115 (18.5) | 51 (19.0) |
|  |  | *Vegetables (salads, cooked vegetables)* | 605 (67.9) | 429 (68.9) | 176 (65.7) |
|  |  | *Fast food* | 51 (5.7) | 33 (5.3) | 18 (6.7) |
|  |  | *Pre-cooked / industrially prepared meal* | 58 (6.5) | 38 (6.1) | 20 (7.5) |
|  |  | *None of these* | 2 (0.2) | 2 (0.3) | 0 |
| Consumption of fruit (including juices made from fresh fruit but excluding juices made from concentrates and canned or dried fruits), n (%) | | *Once or more a day* | 634 (71.2) | 443 (71.1) | 191 (71.3) |
|  |  | *4 to 6 times a week* | 122 (13.7) | 89 (14.3) | 33 (12.3) |
|  |  | *1 to 3 times a week* | 90 (10.1) | 64 (10.3) | 26 (9.7) |
|  |  | *Less than once a week* | 33 (3.7) | 21 (3.4) | 12 (4.5) |
|  |  | *Never* | 12 (1.3) | 6 (1.0) | 6 (2.2) |
| Servings of fruit per day, Med [Q1, Q3] | | | (n=879) | (n=617) | (n=262) |
|  |  |  | 1.0 [1.0;2.0] | 1.0 [1.0;2.0] | 2.0 [1.0;2.0] |
| Consumption of vegetables or salads, excluding potatoes and juices made from concentrates, n (%) | *Once or more a day* | | 499 (56.0) | 351 (56.3) | 148 (55.2) |
|  | *4 to 6 times a week* | | 190 (21.3) | 136 (21.8) | 54 (20.1) |
|  | *1 to 3 times a week* | | 148 (16.6) | 107 (17.2) | 41 (15.3) |
|  | *Less than once per week* | | 39 (4.4) | 19 (3.0) | 20 (7.5) |
|  | *Never* | | 15 (1.7) | 10 (1.6) | 5 (1.9) |
| Servings of vegetables or salads per day, Med [Q1, Q3] | | | (n=876) | (n=613) | (n=263) |
|  |  |  | 1 [1;2] | 1 [1; 2] | 1.0 [1.0;2.0] |
| Consumption of alcoholic beverages of any kind, in the last 12 months,  n (%) | *Every day or almost every day* | | 180 (20.2) | 121 (19.4) | 59 (22.0) |
|  | *5 to 6 days a week* | | 26 (2.9) | 16 (2.6) | 10 (3.7) |
|  | *3 to 4 days a week* | | 35 (3.9) | 28 (4.5) | 7 (2.6) |
|  | *1 to 2 days a week* | | 157 (17.6) | 108 (17.3) | 49 (18.3) |
|  | *2 to 3 days a month* | | 53 (5.9) | 38 (6.1) | 15 (5.6) |
|  | *Once a month* | | 58 (6.5) | 39 (6.3) | 19 (7.1) |
|  | *Less than once a month* | | 81 (9.1) | 63 (10.1) | 18 (6.7) |
|  | *Not consumed in the last 12 months because they stopped drinking* | | 152 (17.1) | 112 (18.0) | 40 (14.9) |
|  | *Never consumed, or only occasionally to taste* | | 149 (16.7) | 98 (15.7) | 51 (19.0) |
| Number of days they usually drink alcohol, between Monday and Thursday, n (%) |  | | (n=398) | (n=273) | (n=125) |
|  | *4 days* | | 190 (47.7) | 126 (20.2) | 64 (23.9) |
|  | *3 days* | | 13 (3.3) | 10 (1.6) | 3 (1.1) |
|  | *2 days* | | 28 (7.0) | 21 (3.4) | 7 (2.6) |
|  | *1 day* | | 60 (15.1) | 45 (7.2) | 15 (5.6) |
|  | *On neither day* | | 107 (26.9) | 71 (11.4) | 36 (13.4) |
| Number of alcoholic drinks consumed, between Monday and Thursday, on average, n (%) |  | | (n=291)^[[1]](#footnote-1)^ | (n=202) | (n=89) |
|  | *16 or more drinks per day* | | 0 | 0 | 0 |
|  | *10 to 15 drinks per da* | | 0 | 0 | 0 |
|  | *6 to 9 drinks per day* | | 4 (1.4) | 4 (0.6) | 0 |
|  | *4 to 5 drinks per day* | | 14 (4.8) | 10 (1.6) | 4 (1.5) |
|  | *3 drinks per day* | | 17 (5.8) | 10 (1.6) | 7 (2.6) |
|  | *2 drinks per day* | | 99 (34.0) | 65 (10.4) | 34 (12.7) |
|  | *1 drink per day* | | 157 (54.0) | 113 (18.1) | 44 (16.4) |
| Number of days they usually drink alcohol, between Friday and Sunday, n (%) |  | | (n=590) | (n=413) | (n=177) |
|  | *3 days* | | 201 (34.1) | 133 (21.3) | 68 (25.4) |
|  | *2 days* | | 110 (18.6) | 76 (12.2) | 34 (12.7) |
|  | *1 day* | | 221 (37.5) | 161 (25.8) | 60 (22.4) |
|  | *On neither day* | | 58 (9.8) | 43 (6.9) | 15 (5.6) |
| Number of alcoholic drinks consumed, between Friday and Sunday, on average, n (%) |  | | (n=532) | (n=370) | (n=162) |
|  | *16 or more drinks per day* | | 0 | 0 | 0 |
|  | *10 to 15 drinks per da* | | 1 (0.2) | 0 | 1 (0.4) |
|  | *6 to 9 drinks per day* | | 10 (1.9) | 6 (1.0) | 4 (1.5) |
|  | *4 to 5 drinks per day* | | 40 (7.5) | 25 (4.0) | 15 (5.6) |
|  | *3 drinks per day* | | 48 (9.0) | 31 (5.0) | 17 (6.3) |
|  | *2 drinks per day* | | 166 (31.2) | 113 (18.1) | 53 (19.8) |
|  | *1 drink per day* | | 267 (50.2) | 195 (31.3) | 72 (26.9) |
| Consumption of 6 or more alcoholic beverages on a single occasion or event, in the last 12 months, n (%) |  | | (n=590) | (n=413) | (n=177) |
|  | *Every day or almost every day* | | 6 (1.0) | 6 (1.0) | 0 |
|  | *5 to 6 days per week* | | 3 (0.5) | 2 (0.3) | 1 (0.4) |
|  | *3 to 4 days per week* | | 4 (0.7) | 2 (0.3) | 2 (0.7) |
|  | *1 to 2 days per week* | | 17 (2.9) | 17 (2.7) | 0 |
|  | *2 to 3 days per month* | | 18 (3.1) | 12 (1.9) | 6 (2.2) |
|  | *Once a month* | | 41 (6.9) | 22 (3.5) | 19 (7.1) |
|  | *Less than once a month* | | 127 (21.5) | 86 (13.8) | 41 (15.3) |
|  | *Not in the last 12 months* | | 186 (31.5) | 134 (21.5) | 52 (19.4) |
|  | *Never in life* | | 188 (31.9) | 132 (21.2) | 56 (20.9) |

**Supplementary Table 2.** Learning group’s univariate logistic regressions to identify factors associated with unhealthy diet. Dependent variable: healthy diet (reference category: “yes”). The variables gender, smoker, screen activity and osteoarticular pain problems in the last 12 months obtained a p-value ≤ 0.20. OR = odds ratio; 95%; CI = 95% confidence interval.

| Variables | | OR [95% CI] | p-value |
| --- | --- | --- | --- |
| Gender | *Female* | *Reference* | |
|  | *Male* | 2.19 [1.13; 4.32] | **0.022** |
| Age | | 1.00 [0.98; 1.02] | 0.820 |
| Marital status | *Married* | *Reference* | |
|  | *Others* | 1.17 [0.62; 2.24] | 0.626 |
| BMI | | 0.97 [0.90; 1.04] | 0.433 |
| Education level completed | *1st cycle or less* | *Reference* | |
|  | *2nd or 3rd cycle* | 1.60 [0.67; 3.85] | 0.293 |
|  | *Secondary* | 1.17 [0.50; 2.74] | 0.719 |
|  | *Higher education* | 1.16 [0.39; 3.44] | 0.788 |
| Main occupation | *Active* | *Reference* | |
|  | *Non-active* | 1.38 [0.71; 2.73] | 0.348 |
| Self-perceived health status | *Very good or good* | *Reference* | |
|  | *Reasonable* | 1.21 [0.62; 2.40] | 0.576 |
|  | *Bad or very bad* | 1.81 [0.51; 7.06] | 0.364 |
| Smoker | *Yes* | *Reference* | |
|  | *No* | 0.22 [0.08; 0.55] | **0.002** |
| Regular physical activity | *No* | *Reference* | |
|  | *Yes* | 0.78 [0.39; 1.55] | 0.483 |
| Screen Activity | *Absent* | *Reference* | |
|  | *Present* | 2.25 [1.10; 4.75] | **0.029** |
| Cardiovascular problems in the last 12 months | *No* | *Reference* | |
|  | *Yes* | 0.71 [0.36; 1.41] | 0.333 |
| Metabolic problems in the last 12 months | *No* | *Reference* | |
|  | *Yes* | 1.56 [0.71; 3.53] | 0.273 |
| Mental health problems in the last 12 months | *No* | *Reference* | |
|  | *Yes* | 1.46 [0.63; 3.48] | 0.382 |
| Osteoarticular pain problems in the last 12 months | *No* | *Reference* | |
|  | *Yes* | 0.60 [0.31; 1.15] | **0.125** |
| No problem in the last 12 months | *No* | *Reference* | |
|  | *Yes* | 1.16 [0.60; 2.26] | 0.659 |

1. The sample size in Supplementary Table 1 varies due to the use of skip patterns, whereby respondents were directed to specific questions, depending on their previous answer. [↑](#footnote-ref-1)
